# Differentiating Pseudohyperkalemia From True Hyperkalemia in a Patient With Chronic Lymphocytic Leukemia and Diverticulitis

**DOI:** 10.7759/cureus.9800

**Published:** 2020-08-17

**Authors:** John Dewey, Joshua Mastenbrook, Laura D Bauler

**Affiliations:** 1 Emergency Medicine, Western Michigan University Homer Stryker M.D. School of Medicine, Kalamazoo, USA; 2 Biomedical Sciences, Western Michigan University Homer Stryker M.D. School of Medicine, Kalamazoo, USA

**Keywords:** pseudohyperkalemia, hyperkalemia, chronic lymphocytic leukemia, elevated potassium, diverticulitis

## Abstract

Acute changes in electrolyte levels can result in severe physiologic complications. Rapid treatment of abnormally elevated potassium levels is essential due to the increased risk of potentially fatal cardiac arrhythmias. However, there are a number of circumstances that can lead to falsely elevated serum potassium levels, including fist clenching during phlebotomy and hemolysis of hematocytes during laboratory processing. Here we present a case of an elderly woman with chronic lymphocytic leukemia who presented with lower left quadrant pain and hematochezia. Laboratory tests revealed an elevated serum potassium level (7.5 mmol/L) on initial testing, in the absence of hyperkalemia symptoms, EKG changes, and hemolysis of the blood specimen. Abdominal CT revealed inflammatory changes consistent with diverticulitis. She was treated with intravenous calcium, insulin, glucose, and bicarbonate for her hyperkalemia and admitted for treatment for diverticulitis. A subsequent serum potassium level (3.9 mmol/L) and discussion with the hospitalist suggested a diagnosis of leukolysis-induced pseudohyperkalemia, and further treatment of hyperkalemia was halted. This case serves to remind current and future physicians about the importance of maintaining clinical suspicion and clarifying unexpected laboratory readings when the clinical picture and results do not completely align.

## Introduction

Hyperkalemia is common in clinical medicine, with an incidence as high as one in ten patients [1]. Treatment of hyperkalemia varies depending on the underlying cause and comorbidities [2]. Untreated hyperkalemia can lead to detrimental outcomes, including potentially fatal cardiac arrhythmias. True hyperkalemia can result from renal dysfunction, endocrinopathies, drug effects, and extreme diet changes [3,4]. Pseudohyperkalemia describes a falsely elevated potassium level upon measurement of the serum electrolyte concentration due to disruption of cells during the collection or processing of the sample [4]. Given the diverse set of causes of hyperkalemia, it is important to recognize when it is in the patient's best interest to administer treatments immediately, and when it is appropriate to re-evaluate. Here we present a case of pseudohyperkalemia in an elderly patient with chronic lymphocytic leukemia (CLL) who presented to the emergency department for left lower quadrant abdominal pain and episodes of bright red blood per rectum and discuss how to differentiate between hyperkalemia and pseudohyperkalemia in an emergency setting.

## Case presentation

An 81-year-old female patient presented to the emergency department (ED) complaining of left lower quadrant abdominal pain and four to five episodes of bright red blood per rectum over the past 24 hours. The patient did not appear to be in any acute distress and denied any current nausea, vomiting, or diarrhea. There were no complaints of muscle weakness, myalgia, numbness, or palpitations. She had a history of hypertension, coronary artery disease, previous stroke, diabetes mellitus, and CLL. She denied any previous gastrointestinal bleeding. The patient’s medication list included ranolazine, losartan, clopidogrel, and aspirin.

The patient’s initial vital signs included a temperature of 97.8˚F, a pulse of 89 bpm, a blood pressure of 165/68 mmHg, a respiratory rate of 22 bpm, and a pulse-ox of 96% on room air. Her physical exam was significant for pale palpebral conjunctiva and abdominal tenderness in the periumbilical region and left lower quadrant with guarding. She did not appear to have pain out of proportion to her exam. There was no Murphy’s sign, Rovsing’s sign, or McBurney’s point tenderness. Bowel sounds were normal, and the abdomen was otherwise soft with no palpable masses. Laboratory tests obtained in the ED showed a marked increase in white blood cells (WBCs) relative to the reference range, consistent with her CLL, and a critically high serum potassium level, among other values (Table 1). 

**Table 1:** Initial Laboratory Values † Denotes values outside of the reference range ‡ Denotes critical lab values BUN, blood urea nitrogen; eGFR, estimated glomerular filtration rate

Value (units)	Result	Reference range
Blood glucose (mg/dL)	402^†^	70-99
Blood urea nitrogen (mg/dL)	29^†^	8-23
Creatinine (mg/dL)	1.31^†^	0.60-1.10
BUN/creatinine ratio	22^†^	6-20
eGFR (mL/min/1.73 m^2^)	39^†^	>60
White blood cell count (x10^9^ cells/L)	115.3^‡^	4.0-11.0
Potassium (mmol/L)	7.5^‡^	3.5-5.3
Sodium (mmol/L)	138	135-145
Chloride (mmol/L)	100	98-108
Bicarbonate (mmol/L)	22	23-32
Anion gap (mmol/L)	16	9-18
Venous pH	7.44	7.32-7.42
Beta-hydroxybutyrate (mmol/L)	0.7^†^	0.02-0.27

**Figure 1:**
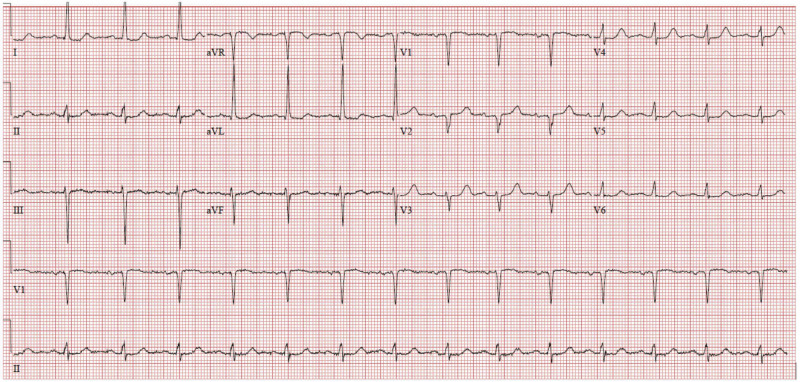
Emergency Department Electrocardiogram. Displaying a normal sinus rhythm with a rate of 87 bpm. The PR interval was 176 msec, QRS duration was 78 msec and QTc was 445 msec. No peaked t-waves were observed.

An EKG revealed a normal sinus rhythm with a rate of 87 beats/minute without peaked T waves or acutely prolonged intervals (Figure 1). 

**Figure 2:**
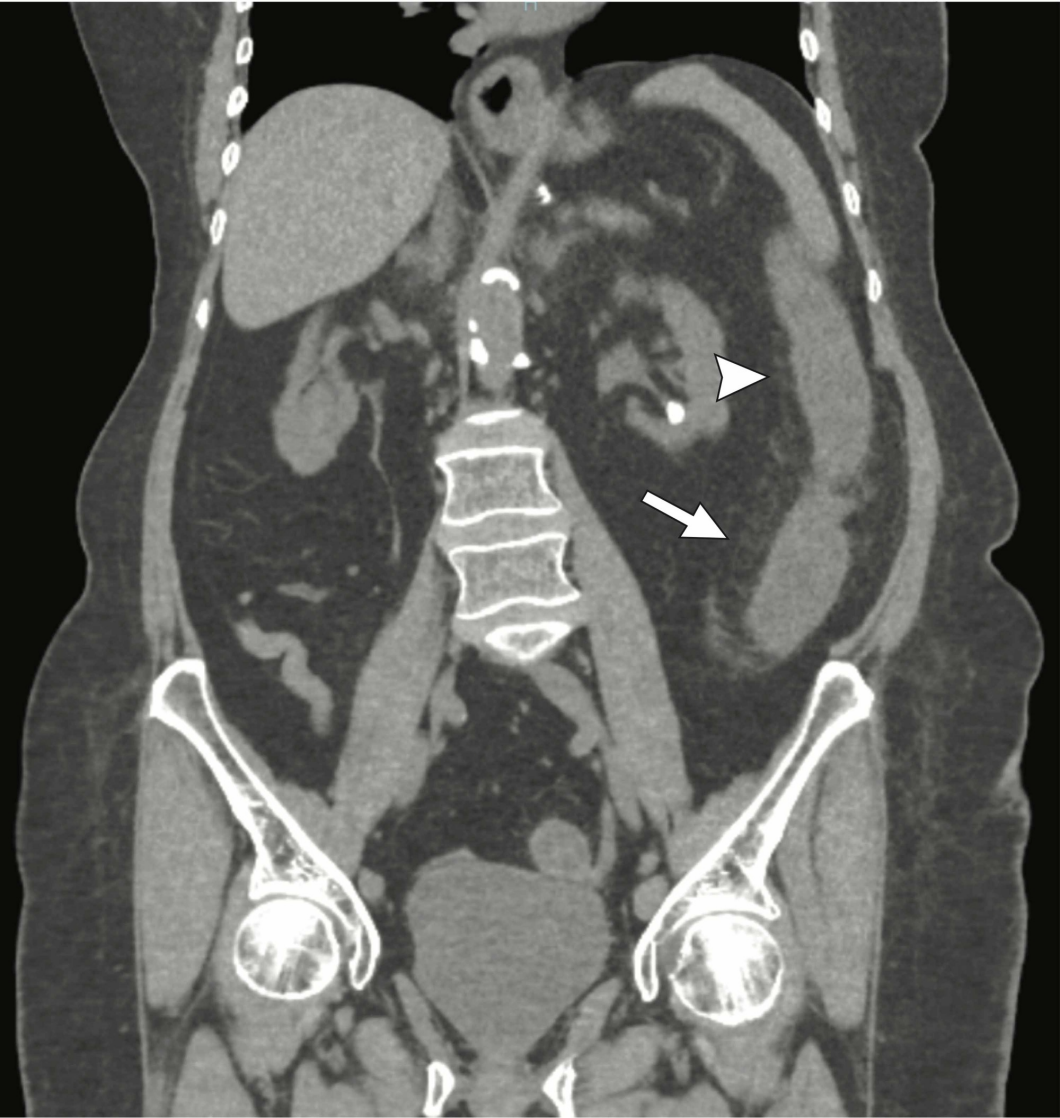
Coronal CT image demonstrating pericolonic inflammatory changes (white arrowhead) and bowel wall thickening (white arrow) of the descending colon.

Abdominal CT imaging showed pericolonic inflammatory change along the course of the left colon extending to the level of the sigmoid colon (Figure 2). 

The hyperkalemia was treated with 10 units of intravenous (IV) regular insulin (Humulin/Novolin R), 15 g of IV dextrose, 2,000 mg calcium gluconate IV piggyback (IVPB), and 50 mEq IV sodium bicarbonate. After the treatment was initiated, she was admitted for management of colitis, hyperkalemia, and reports of gastrointestinal bleeding. Upon further discussion with the internal medicine admission team, there was concern about the lack of EKG changes expected with acute hyperkalemia and the potential complication of lympholysis in the context of CLL. A second serum potassium level was collected approximately 5.5 hours after the initial sample and 2 hours after the initiation of treatment. The new sample was walked to the laboratory without use of the pneumatic tube system, and sampled without centrifugation to minimize potential mechanical lysis of the WBCs. This resulted in a potassium level of 3.9 mmol/L, in contrast to the initial finding of 7.5 mmol/L. Due to the drastic change in potassium and lack of EKG findings, the initial elevated serum potassium level was determined to be an erroneous laboratory finding secondary to mechanical lysis of the excessive lymphocytes. During her hospitalization, she underwent colonoscopy which revealed findings consistent with ischemic colitis from the rectum to the splenic flexure, and no active bleeding. Following a four-day hospitalization and hydration, the patient’s signs and symptoms improved, and she was discharged home and told to hold her dual antiplatelet therapy for one week.

## Discussion

Pseudohyperkalemia is an elevation of potassium in the serum that results during collection, storage, or handling of a blood sample, and is not representative of the potassium level in vivo [5]. Pseudohyperkalemia can result from increased tourniquet tension, increased pressure from a patient’s fist being clenched, or mechanical lysis of hematocytes during pneumatic tube delivery of samples to the laboratory or the centrifugation process [6,7]. Leukolysis can drastically change the reported potassium concentration without recognition that the sample may be hemolyzed, since the red blood cells may remain intact [8]. In the case of CLL, this is more likely due to the increased fragility of the immature lymphocytes.

In this case, the ED physician faced the challenge of differentiating between true hyperkalemia and pseudohyperkalemia. There was no evidence of hemolysis reported by the laboratory. The gastrointestinal hemorrhage served as a potential mechanism of true hyperkalemia. Reabsorption of potassium from the excreted blood could provide a source of the elevated serum potassium level in the patient. WBC tumor lysis in vivo may also have contributed to hyperkalemia, although less likely as the patient had not received chemotherapy recently. Dehydration resulting from the gastrointestinal bleeding would increase the relative concentration of WBCs, which would facilitate a rise in the amount of potassium released from lysis of fragile immature lymphocytes in vitro. The lack of EKG changes associated with such a high potassium level could implicate pseudohyperkalemia. The diagnosis of (lympholysis-induced) pseudohyperkalemia was not determined until the serum potassium level was retested using a more gentle handling process of the sample.

An important distinction between hyperkalemia and pseudohyperkalemia is the presence or absence of symptoms. Hyperkalemia typically presents with muscle weakness or paralysis, and distinct electrical changes on electrocardiograms [9,10]. These changes are typically progressive starting with peaked T waves and continuing through widening of the PR interval, widening of the QRS interval, and eventually loss of the P wave and depression of the ST segment, resulting in a sinusoidal waveform [9,10]. While this is the classic presentation, these steps may not always occur in order, so any one of these changes should heighten suspicion [10]. In our patient, these EKG changes were not observed, although up to 60% of patients with serum potassium levels greater than 6 mmol/L will not have EKG abnormalities [11].

When identifying the cause of true hyperkalemia, assessment of kidney function is essential since a majority of the potassium excreted through the kidneys. Acute kidney injury or underlying chronic kidney dysfunction can result in fluctuations in serum electrolyte levels [12]. Other causes of hyperkalemia to consider include metabolic derangements such as diabetic ketoacidosis (DKA), pharmacologic effects, malignancy, and hemolysis. Pharmacologically, angiotensin-converting enzyme (ACE) inhibitors, angiotensin II receptor blockers (ARBs), potassium-sparing diuretics, beta blockers, calcineurin inhibitors, and heparin use have all been documented to induce hyperkalemia [3,13,14]. The state of high cell turnover found in malignancy can precipitate tumor lysis syndrome, which results in hyperkalemia, hyperphosphatemia, hyperuricemia, and hypocalcemia [15]. Tumor lysis syndrome is predominant in leukemias and lymphomas, especially when receiving chemotherapeutics [16]. Finally, the possibility of a spurious laboratory value must not be dismissed [17]. While the patient’s estimated glomerular filtration rate (eGFR) value suggested abnormal kidney function, it was not significantly altered from her baseline readings, indicating that kidney function was not a likely cause of her hyperkalemia. She did not have DKA based on a venous pH of 7.44 and a normal anion gap, and while she did have CLL, she was not actively being treated, thus limiting the contribution of tumor lysis syndrome in vivo. One factor that may have led to a true state of hyperkalemia was losartan, a diuretic known to alter potassium serum levels. It is also likely that the fragility of her WBC could have contributed to her hyperkalemic laboratory value. While it is possible that the hyperkalemia treatment received in the ED may have reduced potassium levels, it is unlikely that the pharmacologic treatment alone would have resulted in such a drastic change in her serum potassium in that time frame. The onset of sodium bicarbonate hyperkalemia reduction is four to six hours, and the magnitude of that reduction is variable [11]. The onset of insulin induced reduction in serum potassium is 15-20 minutes with an expected reduction of 0.5-1.5 mmol/L.

Malignancy-induced hyperleukocytosis resulting in pseudohyperkalemia has been reported in conjunction with a number of malignancies, including CLL, chronic myeloid leukemia, and T-cell acute lymphoblastic leukemia [18-20]. In each of these cases, the patient had a marked hyperleukocytosis with no apparent clinical signs or symptoms of potassium imbalance. In our case, the challenge was the introduction of a confounding variable, the episodes of gastrointestinal hemorrhage. Since the initial elevated serum potassium value was discovered in the context of an evaluation for abdominal pain and bright red blood per rectum, the electrolyte imbalance was not initially attributed to the underlying CLL but rather to the acutely presenting illness.

For patients with true hyperkalemia, the underlying cause of the imbalance should be investigated and addressed. Immediate medical therapy in the emergent setting typically consists of IV insulin, dextrose, a beta-2 agonist, sodium bicarbonate, and calcium gluconate [11]. Hemodialysis can also be utilized to lower potassium levels in the context of renal failure patients [1]. If renal function is unimpaired, loop and thiazide diuretics can be used to decrease potassium reabsorption [11]. While cation exchangers can be used to limit potassium uptake from the gastrointestinal lumen, their use in the emergency setting is not always indicated [1]. If the cause of a symptomatic hyperkalemia is found to be tied to a pharmaceutical, a medication change should be considered if appropriate [14].

## Conclusions

Blood panels are one of the most informative and commonly used diagnostic tests; thus, errors in these readings may alter the course of treatment for patients. For patients presenting with hyperkalemia, ensuring that the clinical signs and symptoms align with the diagnosis is essential; in cases where the clinical picture does not match, therapy should be held in favor of re-evaluation. This case highlights some of the challenges of addressing electrolyte imbalances in an emergency setting, and how to systematically evaluate a patient in the setting of unexpected laboratory results.
